# Chemistry of the Enaminone of 1-Acetylnaphthalene under Microwave Irradiation Using Chitosan as a Green Catalyst 

**DOI:** 10.3390/molecules16010609

**Published:** 2011-01-17

**Authors:** Huwaida M. E. Hassaneen

**Affiliations:** Department of Chemistry, Faculty of Science, Cairo University, Giza 12613, Egypt; E-Mail: huwaidahassaneen@hotmail.com

**Keywords:** chitosan, 1-acetylnaphthalene, microwave irradiation, enaminone, hydrazonoyl halides

## Abstract

Enaminone **1** was reacted with hydrazonoyl halides **2a-d** to yield 3,4-disubstituted pyrazoles **6a-d**. Coupling with arenediazonium chlorides afforded the 2-(arylhydrazono)-3-(1-naphthalenyl)-3-oxopropionaldehydes **13a-c**. Compounds **13** could be utilized for the synthesis of a variety of arylpyrazoles, arylazolopyrimidines, and pyridazinones *via* reaction with hydrazines, aminoazoles, and active methylene derivatives, respectively. A comparative study of aforementioned reactions was carried out with chitosan as a basic ecofriendly catalyst under conventional heating as well as under pressurized microwave irradiation conditions.

## 1. Introduction

Enaminones are valuable intermediates in synthetic organic chemistry [1,2,3,4]. In the last few years we and others have reported a variety of synthesis of heteroaromatics that have been developed using functionally substituted enamines as readily obtainable building blocks possessing multiple electrophilic and nucleophilic moieties [5,6,7,8,9]. In continuation of this work, the chemistry of enaminone **1** of 1-acetylnaphthalene which can used as precursor in the synthesis of five- and six-membered heterocycles is reported. Furthermore, in light of recent research interest in the utility of green benign approaches in organic synthesis, another aim of the present study was o introduce a new technique by substituting triethylamine and piperidine with an ecologically more acceptable catalyst, e.g., chitosan, a naturally occurring ecofriendly polymer that can be used as a heterogeneous basic catalyst [10,11] and using microwave irradiation as energy source, as the more efficient energetic coupling of solvents with microwave promotes higher rates of temperature increase, which enhance the reaction rates and improve the regioselectivity [12,13,14]. The utility of microwave irradiation in reactions of enaminones have received limited study [15,16].

## 2. Results and Discussion

Condensation of 1-acetylnaphthalene with dimethylformamide dimethylacetal (DMFDMA) afforded enaminone **1** in 89% and 92% yields, respectively, either by refluxing in xylene for 8 h or by heating under MW without solvent at 180 C for 20 min. The same condensation product has been obtained by conventional heating by Kantevari *et al*. [17] and the microwave method is reported in this article (see Experimental section).

**Scheme 1:**
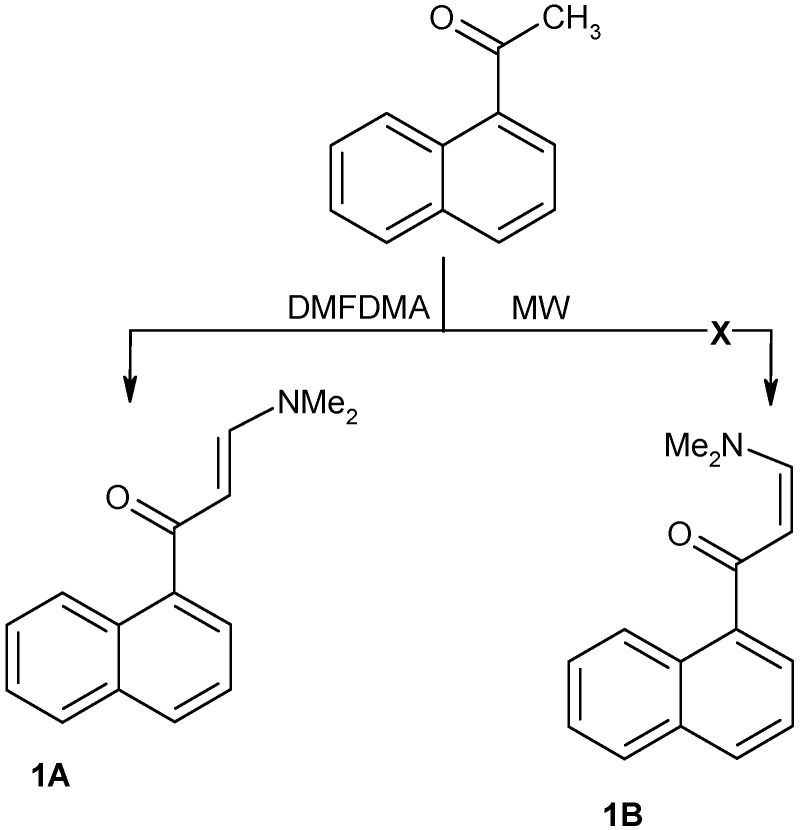
Preparation of 3-dimethylamino-1-naphthalen-1-ylpropenone.

Fischer *et al*. [18] have used coupling of carbonyl groups with protons to establish relative geometry extensively, but the use of ^13^C-NMR to identify the long range coupling of C-H as indication of the stereochemistry with respect to the CO group is very difficult because of appearance of carbonyl carbons as broad signals. We have run NOE difference experiment to establish the stereo-orientation of either the *E* or *Z*-form for enaminone **1A**. The NOE showed that irradiating the alkene CH at δ 5.45 ppm enhanced the dimethylamino protons at δ 2.83 and 3.21 ppm, confirming that they are proximal in space, as required by the *E*-form (Scheme 1).

**Scheme 2:**
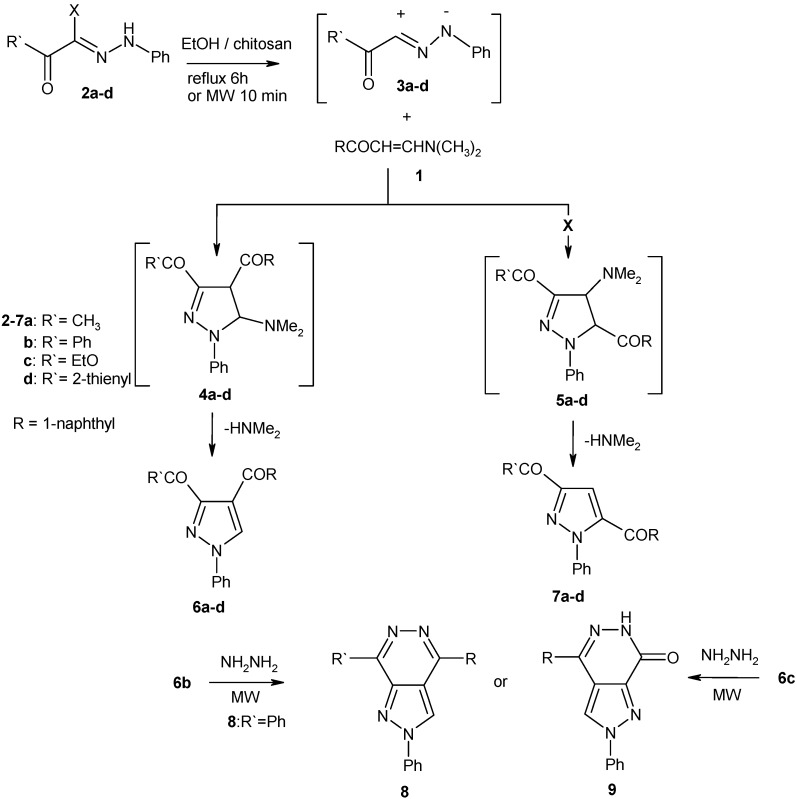
Synthesis of 3,4-dicarbonyl pyrazoles **6** and pyrazolopyridazines **8**, **9**.

The conventional reaction routes of hyrazonoyl halides with dipolarophiles use triethylamine [8,9,19] as basic catalyst to generate, *in situ*, a nitrilimine from the corresponding hydrazonoyl halides. In this context, we used chitosan as a novel ecofriendly basic catalyst in these reactions (chitosan is a copolymer containing both glucosamine units and acetylglucosamine units, the presence of amino groups being responsible for the basic nature of chitosan) [10,11] under microwave irradiation and conventional heating to afford a new environmentally benign route for the synthesis of heterocyclic compounds. 3-Dimethylamino-1-naphthalen-1-ylpropenone (**1)** reacted readily with hydrazonoyl halides **2a-d** in absolute ethanol in the presence of chitosan under microwave irradiation for 10 minutes or reflux for 6 hours to yield the corresponding products of condensation *via* elimination of chitosan hydrochloride. It is assumed that compounds **2** initially furnish nitrilimines **3** which react with **1** to yield either **6** or the isomers **7** *via* loss of dimethylamine from the adducts **4** or **5** (Scheme 2). 

^1^H-NMR and ^13^C-NMR spectra indicated that reaction products are **6a-d** (see Experimental section). The structures of **6b,c** were also confirmed *via* their reaction with hydrazine hydrate. Thus, treatment of **6b,c** with hydrazine hydrate in ethanol (under microwave irradiation or reflux) afforded pyrazolo[3,4-*d*]pyridazine **8** and pyrazolo[3,4-*d*]pyridazin-7-one **9** (Scheme 2). The regiochemistry of addition of **1** to **2** in presence of chitosan under microwave irradiation is similar to the regiochemistry of the reactions of enaminoesters, enaminonitriles, nitroenamines, and enaminones with hydrazonoyl halides **2** [8,9].

**Scheme 3:**
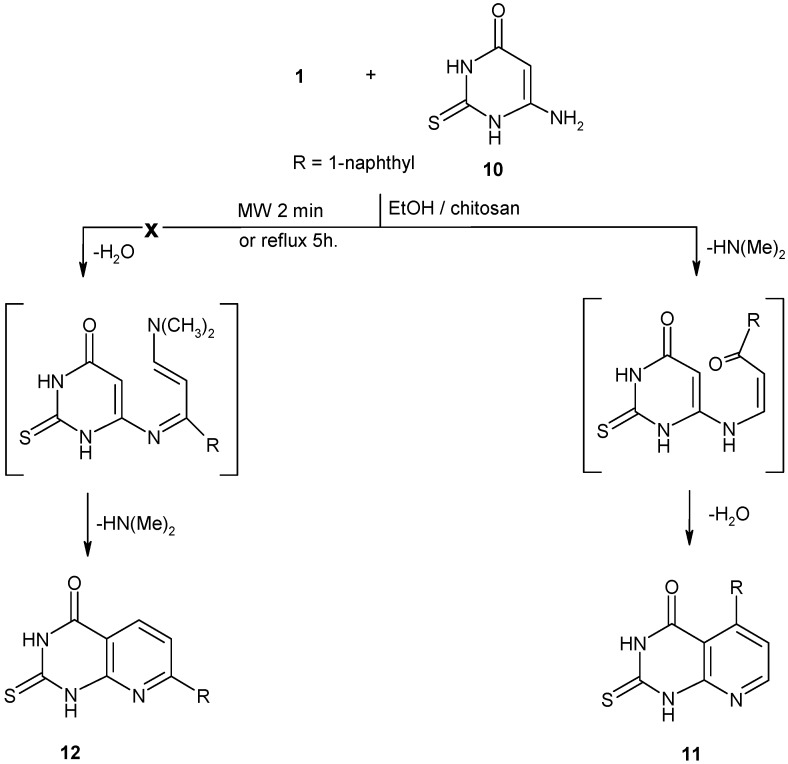
Synthesis of 2-thioxo-pyrido[2,3-d]pyrimidin-4-one (**11**).

We have also investigated the reaction between enaminone **1** and 6-amino-2-thioxo-(1*H*)-pyrimidin-4-one (**10**) in ethanol in the presence of chitosan. This reaction produced 2,3-dihydro-5-(1-naphthalenyl)-2-thioxopyrido[2,3-*d*]pyrimidin-4(1*H*)-one (**11**) or the isomeric structure **12** (Scheme 3). Mass, IR spectra, and elemental analysis data of the isolated product were consistent with both of the isomeric structures **11** and **12** (Scheme 3), while the ^1^H-NMR spectrum revealed a doublet signal at δ 8.42 ppm assigned to a pyridine-2H proton and not a pyridine-4H proton [20], which is consistent with isomeric structure **11**. In addition, according to literature reports the reaction of heterocyclic amines to the double bond of the enaminone occurs with concurrent elimination of dimethylamine rather than condensation of a water molecule [21,22,23]. On the basis of these findings, structure of **12** was discarded and the product isolated from the studied reaction was assigned structure **11** (Scheme 3). 

**Scheme 4:**
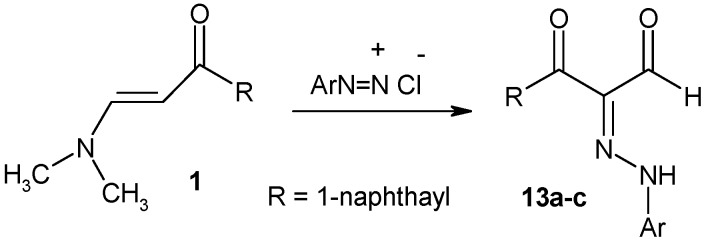
Synthesis of arylhydrazonals **13**.

The chemistry of arylhydrazonals have recently received considerable attention and they serve as useful starting materials in the synthesis of five- and six-membered heterocycles [24]. In this case arylhydrazonals **13a-c** could be readily obtained upon coupling of the enaminone **1** with arenediazonium salts (Scheme 4). 

**Scheme 5:**
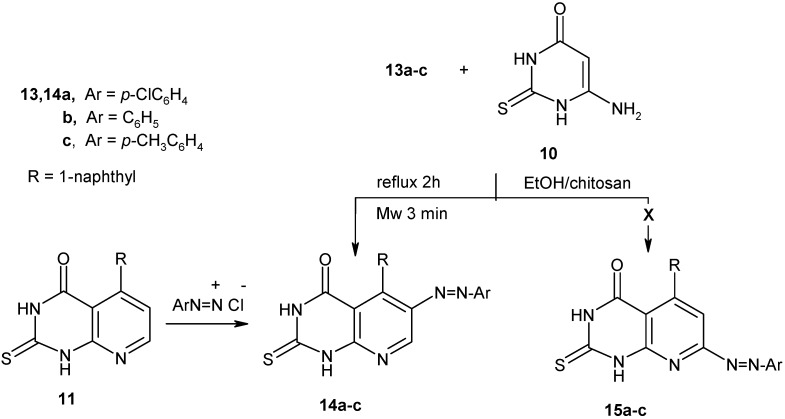
Synthesis of 6-arylazo-2-thioxo-pyrido[2,3-d]pyrimidin-4-ones **14**.

Compound **13a-c** reacted with 6-amino-2-thioxo-(1*H*)-pyrimidin-4-one (**10**) in absolute ethanol in the presence of chitosan (under microwave irradiation or reflux) to afford the 6-arylazo-5-(1-naphthalenyl)-2-thioxo-2,3-dihydro-pyrido[2,3-*d*]pyrimidin-4(1*H*)-ones **14** in excellent yield (92%) and not the isomeric 7-arylazo-5-(1-naphthalenyl)-2,3-dihydro-2-thioxo-pyrido[2,3-*d*]pyrimidin-4(1*H*)-one structures **15** (Scheme 5). The structure assigned to compounds **14a-c** was confirmed by chemical transformation. Thus, coupling of arenediazonium salts with **11** led to formation of products which are identical in all respects (mp, mixed mp, and IR) with products **14a-c** (see Experimental section).

**Scheme 6:**
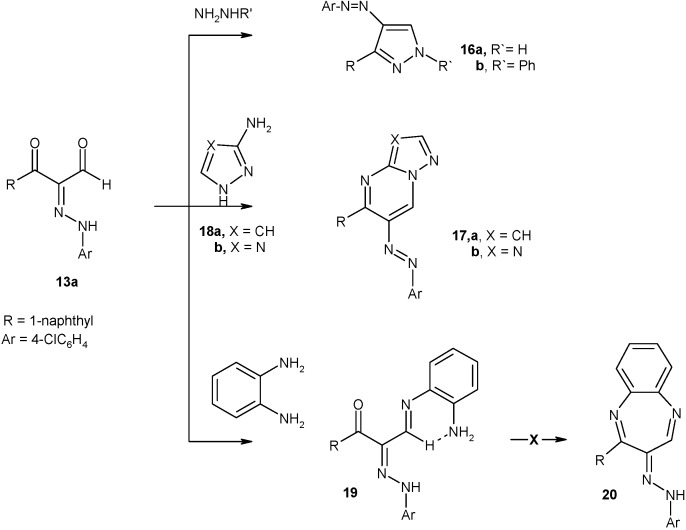
Synthesis of arylpyrazoles, arylazolopyrimidines, and pyridazinones.

An expeditious synthesis of arylpyrazoles **16a,b** and azolopyrimidines **17a,b** by reaction of arylhydrazonal **13a** with hydrazines and aminoazoles **18a,b** proceeded efficiently in ethanol (under microwave irradiation for 5-6 min or reflux for 3 hours) (Scheme 6). *o*-Phenylenediamine also reacted with arylhydrazonal **13a** under the same conditions to give a mono Schiff's base as the uncyclised product **19**, and this compound could not be cyclized to yield benzodiazepine **20** [25], indicating that most likely this product adopted the hydrogen bonded form (Scheme 6).

**Scheme 7:**
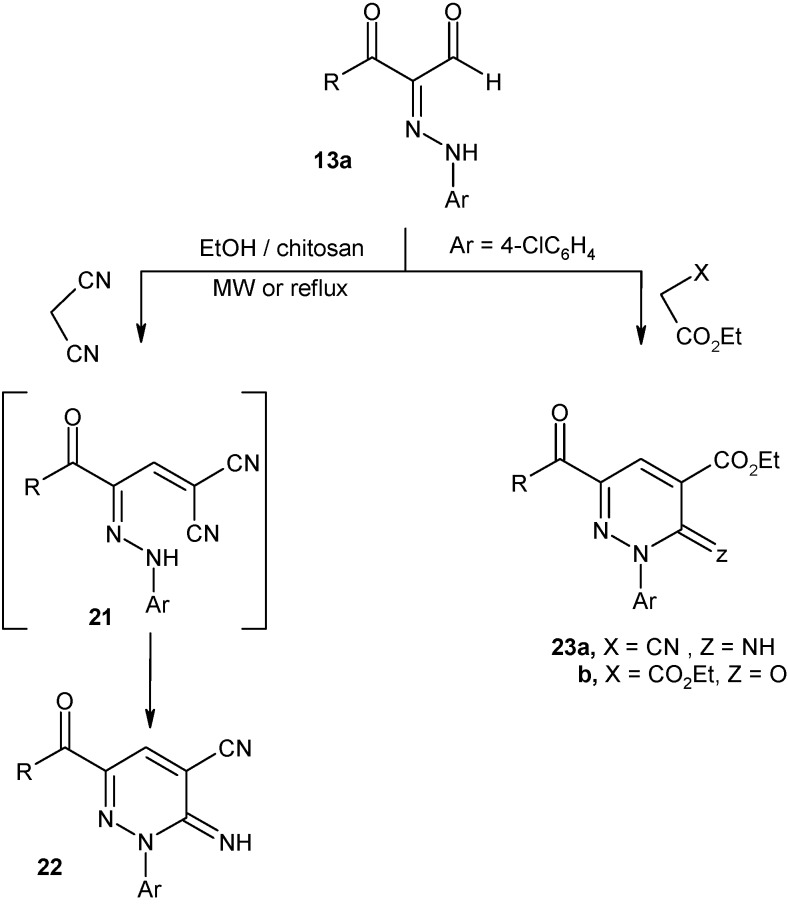
Synthesis of pyridazine derivatives.

2-Arylhydrazonopropanal **13a** condensed readily with malononitrile to yield a product that can be formulated as **21** or the isomeric cyclic **22** (Scheme 7). The structure of **22** was established for the reaction product based on ^13^C-NMR spectroscopy, which revealed only one CN signal at δ = 119. Moreover, the condensation products were stable under conditions reported to effect cyclization of compounds of structure similar to that of **21** [26]. Compound **13a** condensed with ethyl cyanoacetate and diethyl malonate to yield pyridazinones **23a,b** (Scheme 7). 

**Table 1:** Formation of products using pressurized microwave and conventional heating procedures.

Compd. no.	Microwave Irradiation		Conventional Heating	
	Time (min)	Yield (%)	Time (h)	Yield (%)
**6a**	10	80	6	63
**6b**	9	83	6	69
**6c**	9	80	5	66
**6d**	8	87	6	75
**8**	2	89	2	60
**9**	2	87	3	61
**11**	2	84	5	63
**14a**	3	92	2	79
**14b**	3	90	2	78
**14c**	3	90	2	78
**16a**	3	82	3	69
**16b**	3	85	3	72
**17a**	6	77	3	62
**17b**	5	75	3	60
**22**	10	81	5	70
**23a**	10	80	7	65
**23b**	10	79	5	60

A comparative study of the yields of products under pressurized microwave irradiation (method A) and conventional heating (method B) showed that the use of microwave irradiation substantially reduced the reaction times hours scale to minutes and appreciably increased the yields (Table 1).

## 3. Experimental

### 3.1. General

Melting points were determined on an Electrothermal Gallenkamp apparatus and are uncorrected. The IR spectra were recorded in KBr pellets using a Pye Unicam SP-1000 spectrophotometer. ^1^H- NMR spectra (300 MHz) and ^13^C-NMR spectra (75 MHz) were obtained on a Varian EM-300 MH*z* spectrometer using DMSO-d_6_ as solvent with TMS as internal standard. Mass spectra were recorded on an AEI MS 30 mass spectrometer operating at 70 eV. Elemental analyses were carried out by the Microanalytical Center of Cairo University, Giza, Egypt. Microwave experiments were carried out using a CEM Discover Labmate Microwave apparatus (300 W with Chem. Driver software). Compounds **2a** [27], **2b** [28], **2c** [29], **2d** [30] were previously reported.

### 3.2. Preparation of 3-dimethylamino-1-naphthalen-1-ylpropenone ***1***

A mixture of 1-acetylnaphthalene (1.7 g, 10 mmol) and DMFDMA (1.19 g, 10 mmol) was irradiated by focused microwaves at 180 °C for 20 min. The reaction mixture was cooled and purified by flash column chromatography on SiO_2_, eluting with light petroleum-EtOAc (1:1) to afford the pure product **1** as a yellowish brown oil in 92% yield; ^1^H-NMR: δ 2.83 (s, 3H, CH_3_), 3.21 (s, 3H, CH_3_), 5.45 (d, 1H, *J* = 9 Hz), 7.40-7.87 (m, 7H, Ar-H), 8.23 (d, 1H, *J* = 9 Hz); MS *m/z* 225 (M^+^, 10%), 169, 141, 125, 105, 93, 77; Anal; Calcd for C_15_H_15_NO: C, 79.97; H, 6.71; N, 6.22. Found: C, 79.83; H, 6.66; N, 6.18%.

### 3.3. General procedure for preparation of 3-aryl-4-(naphthalene-1-carbonyl)-1-phenyl-1H-pyrazoles ***6a-d***

*Method A*: To a hot solution of **1** (0.22 g, 1 mmol) and the appropriate hydrazonoyl halides **2a-d** (1 mmol) in absolute EtOH (10 mL) was added chitosan (0.1 g). The reaction mixture was irradiated under constant pressure (11.2 Bar, 80 °C) for 8-10 min at power of 300 W. The hot solution was filtered to remove chitosan. After cooling, the reaction mixture was acidified with dil HCl and the solid product was filtered off and crystallized from EtOH to afford the corresponding pyrazole derivatives **6a-d**.

*Method B:* To a hot solution of **1** (2.25 g, 10 mmol) and the appropriate hydrazonoyl halides **2a-d** (10 mmol) in ethanol (25 mL) was added chitosan (1 g). The reaction mixture was refluxed for 5-6 h. The hot solution was filtered to remove chitosan and then the solvent was evaporated under reduced pressure. The solid product was filtered off and crystallized from EtOH to afford the corresponding pyrazole derivatives **6a-d**.

*3-Acetyl-4-(naphthalene-1-carbonyl)-1-phenyl-1H-pyrazole* (**6a**). White crystals; m.p. 163 °C; IR: ν 1643, 1690 (2C=O) cm^-1^; ^1^H-NMR: δ 2.54 (s, 3H, CH_3_CO), 7.41-8.71 (m, 12H, Ar-H), 9.07 (s, 1H, pyrazole-H5); ^13^C-NMR: δ 27.38, 119.45, 124.61, 124.86, 125.49, 126.42, 127.75, 127.92, 128.41, 129.64, 130.14, 132.33, 132.76, 133.43, 135.42, 138.53, 142.65, 150.37, 190.91, 192.87; MS: *m/z* 340 (M^+^, 26.4%), 341, 340, 297, 213, 171, 127, 93, 77, 55; Anal; Calcd for C_22_H_16_N_2_O_2_: C, 77.63; H, 4.74; N, 8.23. Found: C, 77.56; H, 4.66; N, 8.15.%.

*3-Benzoyl-4-(naphthalene-1-carbonyl)-1-phenyl-1H-pyrazole* (**6b**). Yellow crystals; m.p. 165 °C; IR: ν 1651, 1693 (2C=O) cm^-1^; ^1^H-NMR: δ 7.44-8.13 (m, 17H, Ar-H), 9.12 (s, 1H, pyrazole-H5); ^13^C-NMR: δ 118.30, 122.48, 126.65, 127.44, 127.54, 128.09, 128.25, 128.59, 128.65, 128.80, 131.55, 132.31, 132.34, 132.71, 135.15, 135.88, 136.19, 138.55, 138.96, 142.08, 148.34, 177.83, 186.77; MS: *m/z* 402 (M^+^, 12%), 403, 402, 373, 345, 325, 297, 275, 207,155, 127, 105, 77, 55; Anal. Calcd for C_27_H_18_N_2_O_2_: C, 80.58; H, 4.51; N, 6.96. Found:C, 80.52; H, 4.44; N, 6.87.%.

*4-(Naphthalene-1-carbonyl)-1-phenyl-1H-pyrazole-3-carboxylic acid ethyl ester* (**6c**). Pale yellow crystals; m.p. 132 °C; IR: ν 1644, 1715 (2C=O) cm^-1^; ^1^H-NMR: δ 0.97 (t, 3H, *J* = 7.2 Hz, CH_3_), 3.90 (q, 2H, *J* = 7.2 Hz, OCH_2_), 7.45-8.49 (m, 12 H, Ar-H), 9.13 (s, 1H, pyrazole-H5); ^13^C-NMR: δ 14.34, 62.95, 119.35, 120.33, 120.39, 125.29, 127.16, 127.53, 128.30, 130.95, 131.57, 132.40, 133.36, 134.83, 135.25, 135.86, 138.67, 139.58, 145.09, 167.67, 189.74; MS: *m/z* 370 (M^+^, 35%), 371, 370, 341, 325, 297, 247, 215,155, 127, 93, 77, 55; Anal. Calcd for C_23_H_18_N_2_O_3_: C, 74.58; H, 4.90; N, 7.56. Found: C, 74.45; H, 4.84; N, 7.51%.

*4-(Naphthalene-1-carbonyl)-1-phenyl-3-(thienyl-2-carbonyl)-1H-pyrazole* (**6d**). Yellow crystals; m.p. 185 °C; IR: ν 1645, 1697 2(C=O) cm^-1^; ^1^H-NMR: δ 7.24-8.49 (m, 15H, Ar-H), 9.13 (s, 1H, pyrazole-H5); ^13^C-NMR: δ 109.89, 118.51, 119.16, 120.39, 125.81, 127.46, 127.68, 128.35, 131.39, 131.54, 131.70, 132.38, 132.87, 133.46, 135.88, 136.15, 139.45, 139.52, 144.72, 148.11, 151.49, 176.23, 180.23; MS: *m/z* 408 (M^+^, 16%), 409, 408, 379, 363, 347, 297, 281, 242, 207,155, 127, 105, 77, 55; Anal. Calcd for C_25_H_16_N_2_O_2_S: C, 73.51; H, 3.95; N, 6.86; S, 7.85. Found: C, 73.46; H, 3.85; N, 6.82; S, 7.79%.

### 3.4. General procedure for preparation of pyrazolo[3,4-d]pyridazine ***8*** and pyrazolo[3,4-d]pyridazinone ***9***

*Method A:* A mixture of the pyrazoles **6b,c** (1 mmol) and hydrazine hydrate (1 mL) in absolute EtOH (10 mL) was irradiated under constant pressure (11.2 Bar, 80 °C) for 2 min at power of 300 W. The mixture was left to cool and the solid product was collected by filtration and crystallized from DMF.

*Method B:* A mixture of the pyrazoles **6b,c** (10 mmol) and hydrazine hydrate (5 mL) in absolute EtOH (20 mL) was refluxed for 2-3 h. The mixture was left to cool and triturated with ethanol. The solid product was collected by filtration and crystallized from DMF.

*4-Naphthalen-1-yl-2,7-diphenyl-2H-pyrazolo[3,4-d]pyridazine* (**8**). White crystals; m.p. 287 °C; ^1^H- NMR: δ 7.45-8.18 (m, 17H, Ar-H), 8.91 (s, 1H, pyrazole-H5); ^13^C-NMR: δ 120.15, 122.64, 123.12, 123.32, 123.54, 125.87, 126.56, 126.86, 127.35, 127.56, 12887, 128.97, 130.15, 130.32, 130.45, 131.12, 131.68, 132.07, 139.82, 140.35, 141.65, 156.53, 169.65; Anal. Calcd for C_27_H_18_N_4_: C, 81.39; H, 4.55; N, 14.06. Found: C, 81.32; H, 4.48; N, 14.03%.

*4-Naphthalen-1-yl-2-phenyl-2,6-dihydropyrazolo[3,4-d]pyridazin-7-one* (**9**). White crystals; m.p. 292 °C; IR: ν 1670 (C=O), 3136 (NH) cm^-1^; ^1^H-NMR: δ 7.44-8.11 (m, 12H, Ar-H), 8.93 (s. 1H, pyrazole-H5), 12.67 (br., 1H, NH); ^13^C-NMR: δ 119.98, 121.46, 123.29, 123.45, 124.73, 126.05, 126.84, 127.64, 128.57, 129.82, 130.88, 130.93, 131.04, 136.37, 137.87, 139.98, 142.95, 147.22, 165.26; MS: *m/z* 338 (M^+^, 10.7%), 339, 338, 250, 200, 155, 127, 105, 77, 55; Anal. Calcd for C_21_H_14_N_4_O: C, 74.54; H, 4.17; N, 16.56. Found: C, 74.47; H, 4.14; N, 16.52%.

### 3.5. 5-Naphthalen-1-yl-2-thioxo-2,3-dihydro-1H-pyrido[2,3-d]pyrimidin-4-one *(**11**)*

*Method A:* To a mixture of **1** (0.22 g, 1 mmol) and 6-amino-2-thioxopyrimidin-4(1H)-one (**10**, 0.14 g, 1 mmol) in absolute EtOH (10 mL) was added chitosan (0.1 g). The reaction mixture was irradiated under constant pressure (11.2 Bar, 80 °C) for 2 min at power of 300 W. The hot solution was filtered to remove chitosan and after cooling the residual solid was recrystallized from DMF.

*Method B:* A mixture of **1** (2.25 g, 10 mmol) and 6-amino-2-thioxopyrimidin-4(1H)-one (**10**, 1.43 g, 10 mmol) in EtOH (20 mL) was refluxed for 5 h in the presence of chitosan (1 g). The hot solution was filtered to remove chitosan and then the solvent was removed and residual solid was recrystallized from DMF to give yellow crystals m.p. > 300 °C; IR: ν 1687 (C=O), 3479, 3245, 3158 (3NH) cm^-1^; ^1^H-NMR: δ 7.52 (d, 1H, pyridine-H3), 7.55-8.16 (m, 7H, Ar-H), 8.42 (d, 1H, pyridine-H2), 12.62 (br., 1H, NH), 13.17 (br., 1H, NH); ^13^C-NMR: δ 121.61, 124.60, 124.98, 125.08, 125.27, 125.44, 127.15, 127.26, 133.21, 133,93, 138.22, 143.47, 148.80, 149.98, 152.14, 164.15, 170.46; MS: *m/z* 305 (M^+^, 35) 306, 305, 373, 217, 152, 96, 75, 62, 51; Anal. Calcd for C_17_H_11_N_3_OS: C, 66.87; H, 3.63; N, 13.76; S, 10.50. Found: C, 66.81; H, 3.57; N, 13.70; S, 10.46%.

### 3.6. General procedure for preparation of 2-[arylhydrazono]-3-naphthalen-1-yl-3-oxo-propional-dehydes ***13a-c***

A cold solution of arenediazonium salt (10 mmol) was prepared by adding a solution of sodium nitrite (10 mmol) to a cold solution of the corresponding aromatic amine hydrochloride with stirring. The resulting solution of the diazonium salt was added to a cold solution of **1** (2.25 g, 10 mmol), containing sodium acetate. The reaction mixture was stirred at rt for 30 min. The solid product formed was washed with water and crystallized from MeOH.

*2-[(4-Chlorophenyl)hydrazono]-3-naphthalen-1-yl-3-oxo-propionaldehyde* (**13a**). Yellow crystals; m.p. 127 °C; (75% yield); IR: ν 1654, 1677 (2C=O), 3434 (NH) cm^-1^; ^1^H-NMR: δ 7.27-8.17 (m, 11H, Ar-H), 10.03 (s, 1H, CHO), 13.88 (br.,1H, NH); ^13^C-NMR: δ 118.19, 124.51, 125.28, 126.12, 126.94, 128.32, 129.29. 129.58, 130.10, 133.02, 134.24, 135.43, 137.31, 141.08, 146.49, 187.37, 193.49; MS: *m/z* 336 (M^+^, 2.8%), 338, 337, 336, 335, 307, 280, 217, 196, 182, 152, 127, 105, 77, 51; Anal. Calcd for C_19_H_13_ClN_2_O_2_: C, 67.76; H, 3.89; Cl, 10.53, N, 8.32. Found: C, 67.68; H, 3.82; Cl, 10.49, N, 8.30%.

*3-Naphthalen-1-yl-3-oxo-2-(phenylhydrazono)-propionaldehyde* (**13b**). Yellow crystals; m.p. 105 °C; (70% yield); IR: ν 1639, 1680 (2C=O), 3414 (NH) cm^-1^; ^1^H-NMR: δ 7.087-8.21 (m, 12H, Ar-H), 9.84 (s, 1H, CHO), 14.21 (br.,1H, NH); ^13^C-NMR: δ 114.40, 125.59, 126.12, 127.50, 127.98, 130.72, 131.34, 132.13, 132.55, 134.88, 135.43, 135.68, 137.31, 140.90, 141.56, 184.36, 184.34; MS: *m/z* 302 (M^+^, 6.8%), 302, 301, 285, 273, 218, 197, 182, 155, 127, 105, 77, 51; Anal. Calcd for C_19_H_14_N_2_O_2_: C, 75.48; H, 4.67; N, 9.27. Found: C, 75.38; H, 4.58; N, 9.25%.

*2-[(4-Methylphenyl)hydrazono]-3-naphthalen-1-yl-3-oxo-propionaldehyde* (**13c**). Yellow crystals; m.p. 99 °C; (73% yield); IR: ν 1643, 1679 (2C=O), 3409 (NH) cm^-1^; ^1^H-NMR: δ 2.17 (s, 3H, CH_3_) 7.23-8.19 (m, 11H, Ar-H), 9.88 (s, 1H, CHO), 13.89 (br,1H, NH); ^13^C-NMR:) δ 20.87, 117.65, 126.23, 127.54, 127.89, 130.05, 131.17, 132.08, 132.56, 134.75, 134.86, 135.48, 138.65, 136.49, 137.43, 140.96, 184.43, 187.4; MS: *m/z* 316 (M^+^, 9.5%), 317, 316, 301, 287, 232, 217, 197, 182, 155, 127, 107, 91, 77, 51; Anal. Calcd for C_20_H_16_N_2_O_2_: C, 75.93; H, 5.10; N, 8.85. Found: C, 75.82; H, 5.05; N, 8.82%.

### 3.7. General procedure for preparation of pyrido[2,3-d]pyrimidin-4-one ***14a-c***

*Method A*: To a mixture of **13a-c** (1 mmol) and 6-amino-2-thioxopyrimidin-4(1H)-one (**10**, 0.14 g, 1 mmol) in EtOH (10 mL) was added (0.1 g) chitosan at rt. The reaction mixture was irradiated under constant pressure (11.2 Bar, 80 °C) for 3 min. at power of 300 W. The hot solution was filtered to remove chitosan and after cooling the solid formed was recrystallized from DMSO. 

*Method B:* A mixture of **13a-c** (10 mmol) and 6-amino-2-thioxopyrimidin-4(1H)-one (**10**, 1.43 g, 10 mmol) in EtOH (30 mL) was refluxed in the presence of chitosan (1 g) for 2 h. The hot solution was filtered to remove chitosan and then the solvent was removed and residual solid was recrystallized from DMSO. 

*Method C:* A cold solution of arenediazonium salts (10 mmol) was prepared by adding a solution of sodium nitrite (10 mmol) to a cold solution of the aromatic amine hydrochloride with stirring. The resulting solution of the diazonium salt was added to cold solution of **11** (3.05 g, 10 mmol), containing sodium acetate. The reaction mixture was stirred at rt for 30 min. The solid product formed was washed with water and crystallized from DMSO.

*6-(4-Chlorophenylazo)-5-naphthalen-1-yl-2-thioxo-2,3-dihydro-1H-pyrido[2,3-d]pyra-midin-4-one* (**14a**). Orange crystals; m.p. >300 °C; IR: ν 1689 (C=O), 2877, 3052 (2NH) cm^-1^; ^1^H-NMR: δ 7.38-8.12 (m, 11H, Ar-H), 8.59 (s, 1H, pyridine-H), 12.82 (br., 1H, NH), 13.46 (br., 1H, NH); ^13^C-NMR: δ 120.77, 123.67, 123.45, 123.56, 124.09, 125.23, 125.78, 128.65, 131.37, 132.59, 132.86, 134.09, 138.38, 141.44, 148.96, 148.88, 156.78, 157.41, 159.17, 164.45, 168.96; MS: *m/z* 443 (M^+^, 26.4%), 446, 445, 444, 443, 317, 260, 190, 188, 150, 144, 127, 111, 90, 75, 51; Anal. Calcd for C_23_H_14_ClN_5_OS: C, 62.23; H, 3.18; Cl, 7.99; N, 15.78; S: 7.22. Found: C, 62.16; H, 3.12; Cl, 7.90; N, 15.72; S: 7.19%.

*5-Naphthalen-1-yl-6-phenylazo-2-thioxo-2,3-dihydro-1H-pyrido[2,3-d]pyramidin-4-one* (**14b**). Orange crystals; m.p. >300 °C; IR: ν 1717 (C=O), 2881, 3055 (2NH) cm^-1^; ^1^H-NMR: δ 7.37-8.12 (m, 12H, Ar-H), 8.58 (s, 1H, pyridine-H), 12.81 (br., 1H, NH), 13.45 (br., 1H, NH); ^13^C-NMR: δ 120.67, 122.48, 123.51, 123.78, 124.06, 125.18, 125.58, 128.67, 129.34, 129.89, 131.37, 132.56, 132.83, 134.06, 141.34, 147.89, 154.45, 154.76, 156.87, 165.23, 168.99; MS: *m/z* 409 (M^+^, 26.4%), 410, 409, 408, 407, 317, 245, 201, 190, 150, 127, 90, 75, 51; Anal. Calcd for C_23_H_15_N_5_OS: C, 67.47; H, 3.69; N, 17.10; S: 7.83. Found: C, 67.34; H, 3.61; N, 17.04; S: 7.74%.

*5-Naphthalen-1-yl-2-thioxo-6-p-tolylazo-2,3-dihydro-1H-pyrido[2,3-d]pyramidin-4-one* (**14c**). Orange crystals; m.p. >300 °C; IR: ν 1717 (C=O), 2881, 3055 (2NH) cm^-1^; ^1^H-NMR: δ 2.26 (s, 3H, CH_3_), 7.11-8.09 (m, 11H, Ar-H), 8.54 (s, 1H, pyridine-H), 12.71 (br., 1H, NH), 13.32 (br., 1H, NH); ^13^C-NMR: δ 19.42, 111.93, 120.93, 122.29, 122.78, 123.24. 124.90, 125.52, 126.26, 127.93, 128.15, 129.51, 131.18, 132.66, 142.06, 143.03, 149.98, 151.74, 159.49, 162.04, 162.61, 175.94; MS: *m/z* 423 (M^+^, 17.6%), 424, 423, 317, 245, 201, 190, 150, 127, 90, 75, 51; Anal. Calcd for C_24_H_17_N_5_OS: C, 68.07; H, 4.05; N, 16.54; S: 7.57. Found: C, 67.93; H, 3.95; N, 16.45; S: 7.52%.

### 3.8. General procedure for preparation of arylpyrazole derivatives ***16a,b***

*Method A:* A mixture of the **13a** (0.33 g, 1 mmol) and hydrazine derivative (2 mL) in EtOH (10 mL) was irradiated under constant pressure (11.2 Bar, 80 °C) for 3 min at power of 300 W. The reaction mixture was cooled and the solid product was collected by filtration and recrystallized from EtOH. 

*Method B:* A mixture of the **13a** (3.36 g, 10 mmol) and hydrazine derivative (5 mL) in absolute EtOH (20 mL) was refluxed for 3 h. The mixture was left to cool and triturated with EtOH. The solid product was collected by filtration and recrystallized from EtOH. 

*(4-Chlorophenyl)-(4-naphthalen-1-yl-2H-pyrazol-3-yl)-diazene* (**16a**). Yellow crystals; m.p. 203 °C; IR: ν 3334 (NH) cm^-1^; ^1^H-NMR: δ 7.38-8.32 (m, 11H, Ar-H), 8.45 (s, 1H, pyrazole-H5), 11.92 (br.,1H, NH); MS: *m/z* 332 (M^+^, 11.3%), 335, 334, 333, 332, 324, 311, 296, 155, 127, 107, 91, 77, 51; Anal. Calcd for C_19_H_13_ClN_4_: C, 68.57; H, 3.94; Cl, 10.65; N, 16.84. Found: C, 68.50; H, 3.89; Cl, 10.61; N, 16.79%.

*(4-Chlorophenyl)-(3-naphthalen-1-yl-1-phenyl-1H-pyrazol-4-yl)-diazene* (**16b**). Yellow crystals; m.p. 175 °C; ^1^H-NMR: δ 7.24-8.12 (m, 16H, Ar-H), 8.36 (s, 1H, pyrazole-H5); ^13^C-NMR: δ 119.65, 122.56, 124.78, 124.91, 125.67, 126.34, 126.75, 127.09, 127.34, 129.13, 130.08, 132.24, 132.45, 133.12, 136.26, 138.36, 139.66, 140.09, 142.54, 143.68, 154.78; MS: *m/z* 408 (M^+^, 11.3%), 410, 409, 408, 380, 331, 297, 268, 241, 163, 127, 111, 107, 91, 77, 51; Anal. Calcd for C_25_H_17_ClN_4_: C, 73.44; H, 4.19; Cl, 8.67; N, 13.70. Found: C, 73.38; H, 4.14; Cl, 8.61; N, 13.62%.

### 3.9. General procedure for preparation of azolo[1,5-a]pyrimidine derivatives ***17a,b***

*Method A:* A mixture of **13a** (0.33 g, 1 mmol) and the appropriate heterocyclic amine **18a,b** (1 mmol) in EtOH (10 mL) was irradiated under constant pressure (11.2 Bar, 80 °C) for 5-6 min at power of 300 W. The reaction mixture was cooled and the residual solid was recrystallized from acetonitrile to give **17a,b** respectively.

*Method B:* A mixture of **13a** (3.36 g, 10 mmol) and the appropriate heterocyclic amine **18a,b** (10 mmol) in EtOH (20 mL) was refluxed for 3 h. The solvent was removed and residual solid was recrystallized from acetonitrile to give **17a,b** respectively.

*(4-Chlorophenyl)-(5-naphthalen-1-yl-pyrazolo[1,5-a]pyrimidin-6-yl)-diazene* (**17a**). Yellow crystals; m.p. 212 °C; ^1^H-NMR: δ 7.12 (d, 1H, pyrazole-H3) 7.27-8.10 (m, 11H, Ar-H), 8.23 (d, 1H, pyrazole-H2), 9.42 (s, 1H, pyrimidine-H7); MS: *m/z* 383 (M^+^, 12.5%), 385, 384, 383, 356, 328, 295, 273, 258, 218, 203, 188, 164, 139, 111, 90, 75, 51; Anal. Calcd for C_22_H_14_ClN_5_: C, 68.84; H, 3.68; Cl, 9.24, N, 18.25. Found: C, 68.78; H, 3.63; Cl, 9.17, N, 18.18%.

**(***4-Chlorophenyl)-(5-naphthalen-1-yl-[1,2,4]triazolo[1,5-a]pyrimidin-6-yl)-diazene* (**17b**). Yellow crystals; m.p. 242 °C; ^1^H-NMR: δ 7.02-8.10 (m, 11H, Ar-H), 8.48 (s, 1H, triazole-H2), 9.46 (s, 1H, pyrimidine-H7); ^13^C-NMR: δ 117.83, 120.53, 124.49, 125.40, 126.04, 126.24, 126.70, 127.39, 128.28, 129.44, 129.68, 130.23, 130.34, 132.66, 132.93, 136.07, 154.36, 155.80, 168.55; MS: *m/z* 384 (M^+^, 12.5%), 386, 385, 384, 357, 329, 295, 273, 258, 218, 203, 188, 164, 139, 111, 90, 75, 51; Anal. Calcd for C_21_H_13_ClN_6_: C, 65.54; H, 3.41; Cl, 9.21, N, 21.84. Found: C, 65.50; H, 3.38; Cl, 9.18, N, 21.80%.

*3-(2-Aminophenylimino)-2-[(4-chlorophenyl)-hydrazono]-1-naphthalen-1-yl-propan-1-one* (**19**). A mixture of **13a** (0.33 g, 1 mmol) and *o*-phenylenediamine (1 mmol) in EtOH (10 mL) The reaction mixture was irradiated under constant pressure (11.2 Bar, 80 °C) for 10 min at power of 300 W and then poured on to crushed ice. The separated solid was filtered, washed with water and crystallized from acetonitrile to give yellow crystals; m.p. 244 °C; (80% yield); IR (KBr) 1635 (C=O), 3155, 3333, 3354 (NH, NH_2_) cm^-1^; ^1^H NMR (DMSO*-d_6_*) δ 6.99-8.14 (m, 16H, Ar-H), 11.82 (br., 1H, NH), 12.87 (br., 2H, NH_2_); ^13^C NMR (DMSO-*d_6_*) δ 116.72, 117.97, 118.55, 124.34, 125.43, 126.78, 127.57, 127.89, 128.24, 128.66, 128.98, 130.98, 131.19, 131.76, 134.85, 135.22, 135.65, 138.75, 140.48, 141.89, 126.04, 147.14, 186.19; MS: *m/z* 426 (M^+^, 65.7%), 428, 427, 426, 408, 395, 286, 270, 253, 207, 167, 144, 127, 111, 90, 75, 51; Anal. Calcd for C_25_H_19_ClN_4_O: C, 70.34; H, 4.49; Cl, 8.30; N, 13.12. Found: C, 70.27; H, 4.43; Cl, 8.24; N, 13.10%.

### 3.10. General procedure for preparation of pyridazine derivatives ***22*** and ***23a,b***

*Method A*: To a suspension of **13a** (0.33g, 1 mmol) in EtOH (10 mL) and malononitrile, or ethyl cyanoacetate, or diethyl maolnate (1 mmol) was added chitosan (0.1 g) at rt. The reaction mixture was irradiated under constant pressure (11.2 Bar, 80 °C) for 10 min at power of 300 W. The hot solution was filtered to remove chitosan. The reaction mixture was cooled and poured into water. The solid product, so formed, was collected by filtration and recrystallized from acetonitrile.

*Method B:* To a suspension of **13a** (3.36 g, 10 mmol) in EtOh (30 mL) and malononitrile, ethyl cyanoacetate, and diethyl maolnate (10 mmol), chitosan (1 g) was added. The mixture was refluxed for 5-7h. The hot solution was filtered to remove chitosan, left to cool to rt and then poured into water. The solid product, so formed, was collected by filtration and recrystallized from acetonitrile.

*2-(4-Chlorophenyl)-3-imino-6-(naphthalene-1-carbonyl)-2,3-dihydropyridazine-4-carbonitrile* (**22**). Brown crystals; m.p. 150 °C; IR: ν 1646 (C=O), 2228 (CN), 3430 (NH) cm^-1^; ^1^H-NMR: δ 3.07 (br., 1H, NH) 7.27-8.42 (m, 12H, Ar-H, H-5); ^13^C-NMR: 119.32, 121.78, 125.95, 127.34, 127.53, 127.87, 128.44, 129.54, 130.70, 132.32, 133.12, 134.10, 135.15, 136.18, 137.39, 142.58, 142, 81, 146.94, 151.72, 187. 95; MS: *m/z* 384 (M^+^, 12.5%), 386, 385, 384, 383, 348, 286, 258, 216, 190, 163, 127, 111, 90, 75, 51; Anal. Calcd for C_22_H_13_ClN_4_O: C, 68.67; H, 3.41; Cl, 9.21, N, 14.56. Found: C, 68.59; H, 3.36; Cl, 9.17, N, 14.50. %.

*2-(4-Chlorophenyl)-3-imino-6-(naphthalene-1-carbonyl)-2,3-dihydropyridazine-4-carboxylic acid ethyl ester* (**23a**). Yellow crystals; m.p. 165 °C; IR: ν 1626, 1701 (2C=O), 3420 (NH) cm^-1^; ^1^H-NMR: δ 1.35 (t, 3H, *J* = 7.2 Hz, CH_3_), 4.49 (q, 2H, *J* = 7.2 Hz, OCH_2_) 7.27-8.54 (m, 13H, Ar-H, H5, NH); ^13^C-NMR: δ 13.94, 61.73, 126.26, 127.55, 127.95, 128.45, 129.87, 130.74, 130.88, 131.27, 132.54, 143.16, 134.89, 136.34, 137.39, 140.87, 142.41, 142.49, 147.26, 147.29, 159.37, 187.96; MS: *m/z* 431 (M^+^, 12.5%), 433, 432, 431, 350, 286, 258, 216, 190, 163, 127, 111, 90, 75, 51; Anal. Calcd for C_24_H_18_ClN_3_O_3_: C, 66.75; H, 4.20; Cl, 8.21, N, 9.73. Found: C, 66.67; H, 4.12; Cl, 8.15, N, 9.71%.

*2-(4-Chlorophenyl)-6-(naphthalene-1-carbonyl)-3-oxo-2,3-dihydropyridazine-4-carboxylic acid ethyl ester* (**23b**). Yellow crystals; m.p. 159 °C; IR: ν 1632, 1649, 1726 (3C=O) cm^-1^; ^1^H-NMR: δ 1.25 (t, 3H, *J* = 7.2 Hz, CH_3_), 4.31 (q, 2H, *J* = 7.2 Hz, OCH_2_) 7.29-8.19 (m, 11H, Ar-H, NH), 8.29 (s, 1H, H-5); ^13^C-NMR: δ 13.95, 60.16, 120.68, 125.45, 127.14, 127.34, 127.56, 129.85, 130.75, 133.19, 134.15, 134.87, 135.18, 135.45, 136.22, 140.72, 141.46, 143.34, 151.43, 156.87, 164.95, 189.75; MS: *m/z* 432 (M^+^, 12.5%), 434, 433, 432, 383, 348, 286, 258, 216, 190, 163, 127, 111, 90, 75, 51; Anal. Calcd for C_24_H_17_ClN_2_O_4_: C, 66.59; H, 3.96; Cl, 8.19, N, 6.47. Found: C, 66.53; H, 3.91; Cl, 8.12, N, 6.44%.

## 4. Conclusions

It was demonstrated that the enaminone of 1-acetylnaphthalene **1** is a readily obtainable andversatile reagent which can be used for the synthesis of polyfunctional and condensed five- and six-membered heterocycles and also that the microwave-assisted process, in contrast to conventional heating, gave the desired products in higher yield with shorter reaction times. Under pressurized microwaves, solvents can be heated up to temperatures 2 to 4 times higher than their respective boiling points which enhance the reaction rates. Expanding the scope for utilizing chitosan as basic catalyst was investigated in these reactions.
